# Effects of the pulsed electromagnetic field PST® on human tendon stem cells: a controlled laboratory study

**DOI:** 10.1186/s12906-016-1261-3

**Published:** 2016-08-18

**Authors:** Pietro Randelli, Alessandra Menon, Vincenza Ragone, Pasquale Creo, Umberto Alfieri Montrasio, Carlotta Perucca Orfei, Giuseppe Banfi, Paolo Cabitza, Guido Tettamanti, Luigi Anastasia

**Affiliations:** 1 IRCCS Policlinico San Donato, San Donato Milanese, Milan, Italy; 2 Department of Biomedical Sciences for Health, University of Milan, piazza Malan 2, 20097 San Donato Milanese Milan, Italy; 3 IRCCS Istituto Ortopedico Galeazzi, Milan, Italy; 4 Università Vita-Salute San Raffaele, Milan, Italy

**Keywords:** Tendon stem cells, Pulsed electromagnetic fields, Pulsed signal therapy, Rotator cuff

## Abstract

**Background:**

Current clinical procedures for rotator cuff tears need to be improved, as a high rate of failure is still observed. Therefore, new approaches have been attempted to stimulate self-regeneration, including biophysical stimulation modalities, such as low-frequency pulsed electromagnetic fields, which are alternative and non-invasive methods that seem to produce satisfying therapeutic effects. While little is known about their mechanism of action, it has been speculated that they may act on resident stem cells. Thus, the purpose of this study was to evaluate the effects of a pulsed electromagnetic field (PST®) on human tendon stem cells (hTSCs) in order to elucidate the possible mechanism of the observed therapeutic effects.

**Methods:**

hTSCs from the rotator cuff were isolated from tendon biopsies and cultured in vitro. Then, cells were exposed to a 1-h PST® treatment and compared to control untreated cells in terms of cell morphology, proliferation, viability, migration, and stem cell marker expression.

**Results:**

Exposure of hTSCs to PST® did not cause any significant changes in proliferation, viability, migration, and morphology. Instead, while stem cell marker expression significantly decreased in control cells during cell culturing, PST®-treated cells did not have a significant reduction of the same markers.

**Conclusions:**

While PST® did not have significant effects on hTSCs proliferation, the treatment had beneficial effects on stem cell marker expression, as treated cells maintained a higher expression of these markers during culturing. These results support the notion that PST® treatment may increase the patient stem cell regenerative potential.

**Electronic supplementary material:**

The online version of this article (doi:10.1186/s12906-016-1261-3) contains supplementary material, which is available to authorized users.

## Background

Rotator cuff tendinopathy is a degenerative process causing pain and disability [1, 2]. Generally, a non-operative management is regarded as the first-line treatment prior to a surgical intervention [3]. Nonetheless, given the rather inconsistent results of either approaches, new strategies have been explored, especially in the rising field of tissue engineering [4–10]. The discovery that also the human rotator cuff tendons possess a reservoir of progenitor cells, the human tendon stem cells (hTSCs), has clearly opened up new perspectives for developing novel therapies [11–15]. In fact, hTSCs could be used in cell therapy approaches and, maybe most importantly, they could potentially be activated in situ, in order to regenerate the affected tendon without the need of harvesting them from biopsies, growing them in vitro, and re-injecting them. Actually, growing evidences seem to support the notion that when adult stem cells are injected to regenerate a tissue, like the heart or a tendon, only a negligible fraction of cells can survive and differentiate into the desired cell phenotype [16]. In fact, the majority of the injected stem cells simply secrete factors (most of them still unknown) that stimulate resident progenitor cells, which are really the ones that repair the affected tissue. Therefore, in the last few years, researchers have focused their attention on identifying these secreted factors, to use them instead of injecting cells [16–19]. On the other hand, other approaches have been attempted to stimulate self-regeneration, including biophysical stimulation modalities, such as low-frequency pulsed electromagnetic fields (PEMFs), which are alternative and non-invasive methods that seem to produce satisfying therapeutic effects also on a wide range of orthopedics diseases, including osteoarthritis [20, 21], non-united fractures [22, 23], failed arthrodesis [24], and soft tissue injuries [25]. However, little is known about the real mechanism by which these effects are obtained. A plausible explanation is that these electromagnetic fields can stimulate the self-healing processes of the affected tissues by activating their resident stem cells. Among others, a specific low-frequency pulsed electromagnetic field (commercially known as PST®, by Global Munich Germany) is a modification of other low frequency PEMFs, and it has been developed to ideally correspond to the body’s own stimulatory energy parameters. The PST® device consists in a cylindrical solenoid in which the patient can insert the affected area (including the shoulder) during treatment. It employs direct current with unidirectional low frequencies in the range of 10–30 Hz. The waveform is quasi-rectangular, with measured field strengths predominantly in the 0.5–1.5 mT range. Various frequency-amplitude combinations are automatically switched over and transmitted under continuous control during the treatment period, which is generally set to 1 h.

Based on these premises, the aim of this study was to evaluate the effects of PST® on primary cultures of hTSCs and to test whether the application of this pulsed electromagnetic field could alter their phenotype, for instance by increasing their stemness and/or their regenerative capacity, with the ultimate goal of clarifying the mechanism of the observed therapeutic effects.

## Methods

### Isolation, characterization, and culture of human tendon stem cells

**Fig. 1:**
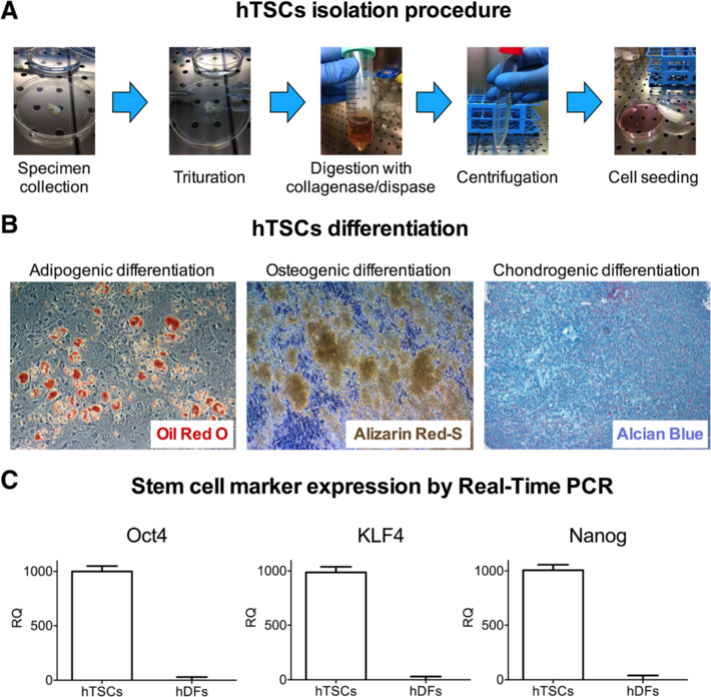
Isolation and characterization of human tendon stem cells (hTSCs). **a** Schematic representation of the protocol used to isolate hTSCs. **b** In vitro differentiation of hTSCs toward the adipogenic, the osteogenic, and the chondrogenic phenotypes. Lipid intracellular droplets (*red*) in the adipocytes were stained with Oil Red O solution. Alizarin Red-S staining revealed the presence of calcium deposits 2016 (*yellowish*-*brown*). Alcian Blue staining detected/assessed the proteoglycan content. Typical results are shown. Original magnification x10. **c** Gene expression of stem cell marker (Oct4, KLF4, and Nanog) by Real-Time PCR in hTSCs and human dermal fibroblasts (hDFs). Data are expressed as means ± SD of three different experiments

Human tendon stem cells (hTSCs) were isolated from supraspinatus tendon specimens collected during arthroscopic rotator cuff repair, according to a previous procedure (Fig. 1a) [11]. The study protocol has been approved by the Hospital Ethical Committee (authorization number 2642; Sept 19, 2011; ASL Milano 2, Melegnano, Milan) and patients received and signed an informed consent. Briefly, samples from supraspinatus tendons (4–8 mm wide) were collected from six patients, kept in HypoThermosol (BioLife Solutions) at 4 °C, and processed separately within 24 h, according to the procedure described below. Samples were washed with phosphate-buffered saline (PBS) (Euroclone), cut into small pieces, and digested for 90 min with collagenase type I (3 mg/mL; Worthington) and dispase (4 mg/mL; Gibco, Life Technologies) in PBS at 37 °C. After centrifugation, cell pellets were resuspended in the following culture medium: α-Minimal Essential Medium (α-MEM) (Sigma-Aldrich) supplemented with 2 mM glutamine (Euroclone), 1 % antibiotic-antimycotic mixture (Euroclone) and 20 % (v/v) fetal bovine serum (FBS) (HyClone, Thermo-Fisher Scientific). Cells were then filtered with a cell strainer (70 mM; BD Falcon) and plated in 150-cm^2^ dishes. Adherent cells were cultured at 37 °C with a humidified atmosphere of 5 % CO_2_. The medium was changed every 2–3 days. The isolated hTSCs at passage three were characterized by flow cytometry for the expression of key stem cell markers and to test the level of contamination with other cell types (Additional file 1: Figure S1). Flow cytometry analysis was performed on 1 × 10^5^ cells/sample. Briefly, aspecific binding sites were blocked with a blocking solution (50 % 1x PBS, 50 % FBS) for 30 min at room temperature, and washed twice with PBS. Cells were stained with fluorochrome-conjugated mouse anti-human antibodies at the optimal concentration (1:20 dilution) in PBS for 10 min at 4 °C, and washed twice with PBS at 4 °C. Cell characterization was performed using the following antibodies: αCD9 FITC, αCD73 FITC, αHLA-DR FITC, αCD13 PE, αCD29 PE, αCD44 PE, αCD45 PE, αCD90 PE, αCD105 PE, αCD106 PE, αCD34 PerCP-eFluor710, αCD166 PerCP-eFluor710, and αSSEA-4 PE (all from eBioscience); αLineage Cocktail FITC, αCD18 PE, αCD146 PE, and αStro-1 Alexa Fluor 647 (all from BioLegend); and αCD117 PE (Miltenyi Biotech). The respective isotype antibodies were used as controls. Samples were acquired with a Navios flow cytometer (Beckman Coulter), and data were processed with Kaluza 1.2 software (Beckman Coulter). Cells at passage three were used for all the experiments.

### Cell differentiation

hTSCs were induced to differentiate toward adipocytes, osteoblasts, and chondroblasts in vitro to test for their multi-lineage potential, according to the following procedures (Fig. 1b).

#### Adipogenic differentiation

hTSCs were plated at a concentration of 3 × 10^4^ cells/cm^2^ in normal growth medium, and then switched to DMEM-low glucose (Sigma-Aldrich), 10 % FBS (HyClone, Thermo-Fisher Scientific), 4 mM L-glutamine (Euroclone), 1 % antibiotic-antimycotic mixture (Euroclone), with the addition of the mesenchymal stem cell adipogenesis kit (Millipore) for 21 days, according to the manufacturer’s instructions. At day 21, Oil Red O solution (Millipore) was used to stain lipid droplets of derived adipocytes, according to the manufacturer’s procedures. All photomicrographs were acquired with an Axiovert 40 microscope (Zeiss) equipped with a Moticam 2300 camera (Motic). The adipogenic medium was changed every 2–3 days.

#### Osteogenic differentiation

hTSCs were plated at a concentration of 3 × 10^4^ cells/cm^2^ in normal growth medium, and then switched to the osteogenesis induction medium, which was constituted of DMEM-low glucose (Sigma-Aldrich), 10 % FBS (HyClone, Thermo-Fisher Scientific), 4 mM L-glutamine (Euroclone), 1 % antibiotic-antimycotic mixture (Euroclone), supplemented with 0.1 μM dexamethasone, 50 μg/ml L-ascorbic acid-2-phosphate, and 10 mM β-glycerophosphate (all reagents from Sigma-Aldrich) for 17 days. At day 17, Alizarin Red solution (Millipore) was used to detect calcium deposition in derived osteoblasts, according to the manufacturer’s instruction. All photomicrographs were acquired with an Axiovert 40 microscope (Zeiss) equipped with a Moticam 2300 camera (Motic). The osteogenic medium was changed every 2–3 days.

#### Chondrogenic differentiation

hTSCs were maintained in a 3D culture by growing them in cell pellets (1 × 10^6^ cells/pellet) in AdvanceSTEM chondrogenic differentiation medium (HyClone, Thermo Scientific), according to the manufacturer’s instructions. After 28 days of differentiation, matrix deposition by derived chondroblasts was detected with Alcian Blue staining (Sigma-Aldrich), according to the manufacturer’s instruction. All photomicrographs were acquired with an Axiovert 40 microscope (Zeiss) equipped with a Moticam 2300 camera (Motic). The chondrogenic medium was changed every 2–3 days.

### PST® device and cell treatments

**Fig. 2:**
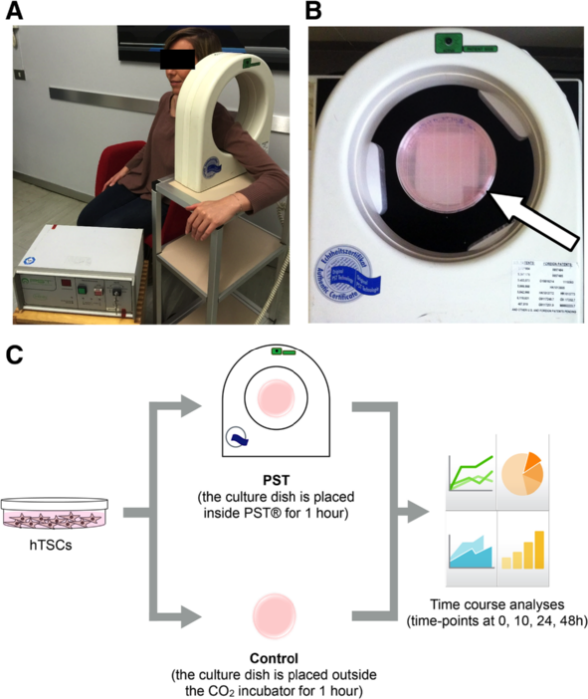
Pulsed Signal Therapy® (PST®). **a** A typical setting for PST® treatment of patients with rotator cuff tendinopathy. **b** A typical experimental setting for PST® treatment of hTSCs. The *white arrow* points to the culture dish positioned at the center of the solenoid. **c** Schematic representation of the experimental setup: twenty-four hours after seeding, hTSCs were divided into two groups, either treated with PST® for 1 h (PST) or kept outside the incubator for the same amount of time (control). Then, PST and control cells were returned to the CO_2_ incubator and cultured for 10, 24, and 48 h for successive analyses

The PST® treatment was performed by placing the cell culture plate at the center of PST® device in order to have the magnetic field vector perpendicular to the plate surface (Fig. 2). hTSCs were plated at a concentration of 2.6 × 10^3^ cells/cm^2^ in normal growth medium. Twenty-four hours after seeding, cells were either treated with PST® for 1 h (PST), or were kept outside the incubator for the same amount of time (control) (Fig. 2c). PST and control cells were then returned to the incubator and grown for other 10, 24, or 48 h.

### Cell morphology and proliferation experiments

To assess whether PST® stimulation could affect hTSCs phenotype, cell morphology was examined with a phase-contrast microscope (Axiovert 40 CFL, Zeiss, equipped with a Moticam 2300 camera, Motic) after 0, 10, 24, and 48 h of PST® exposure. For cell viability analyses, hTSCs were subjected to PST® stimulation, as described before. PST and control cells were analyzed at each time point after harvesting with Trypsin-EDTA solution (Sigma-Aldrich) by counting with a Countess Cell Counter (Invitrogen, Life Technologies), according to the manufacturer’s procedure. Cell viability was determined by trypan blue dye exclusion assay. The number of viable cells in each sample was expressed as a percentage of the total untreated cells number at day 0. All assays were carried out in triplicates for each sample.

### Cell viability by MTT assay

hTSCs were plated in 12-well plates (1 × 10^4^ cells/well) and were subjected to PST® stimulation, as previously described. At each time point (0, 10, 24, and 48 h), two hours before collection, the reconstituted 3-(4,5-dimethylthiazol-2-yl)-2,5-diphenyl-2H-tetrazolium bromide (MTT) (5 mg/ml in PBS; Sigma) was added to the medium (10 % of the final volume). Following a 2-hour incubation at 37 °C, PST and control cells were lysed by adding an amount of MTT Solubilization Solution equal to the original culture medium volume, gently pipetting to completely dissolve the MTT formazan crystals. The MTT reduction was spectrophotometrically measured at a wavelength of 570 nm.

### Cell migration by wound-healing assay

Wound-healing assay was performed as previously described [26]. hTSCs were grown to confluence in 6-well plates and were subjected to PST® stimulation or were kept outside the incubator for the same amount of time (controls). A sterile P200 pipet tip was used to create a scratch across the cell monolayer. Then, cultures were washed once with 1 ml of growth medium to remove the damaged and detached cells. After replacing the medium, hTSCs were allowed to grow for 48 h. At different time points, cell cultures were examined with a phase-contrast microscope (Axiovert 40 CFL, Zeiss, equipped with a Moticam 2300 camera, Motic) and images of the same scratch fields were acquired at time 0 and after 5, 20, 24, and 30 h from the scratch. The gap area between the cells was calculated in each acquired image using software ImageJ. The migration rate was based on the measure of the recovered wound area (experimental data expressed in percentage). All assays were carried out in triplicates for each sample.

### Cell apoptosis analysis

Apoptosis was measured by flow cytometry on PST and control cells before a 1-h PST® treatment and then 10, 24 and 48 h post treatment, using Annexin V-FITC Apoptosis detection kit (Enzo Life Sciences), according to the Manufacturer’s protocol. Briefly, adherent cells were trypsinized, washed in PBS by gentle shaking, and resuspended with 200 μl of a specific Binding Buffer (10 mM HEPES/NaOH, pH 7.4; 140 mM NaCl; 2.5 mM CaCl_2_) containing 5 μl of annexin V-FITC. After incubation for 10 min in the dark at room temperature, cells were washed in PBS, resuspended in 190 μl of Binding Buffer, and then stained with 10 μl Propidium Iodide (20 μg/ml). Samples were acquired with a Navios flow cytometer (Beckman Coulter), and analyzed using Kaluza 1.2 software (Beckman Coulter).

### Gene expression analysis

Stem cell, tendon-related marker, and vascular endothelial growth factor expression was tested by Real-Time PCR. Total RNA was extracted from PST and control cells at 0, 10, 24 and 48 h using TRIzol®Reagent (Ambion, Life Technologies) and 1 μg of extracted RNA was reverse transcribed to cDNA using the iScript cDNA synthesis kit (BioRad), according to the Manufacturer’s instructions. Real-Time PCR was performed in a 96-well plate with 10 ng of cDNA as template, 0.2 μM primers, and 1× Power SYBR® Green PCR Master Mix (Applied Biosystems, Life Technologies) in a 20 μl final volume per well, using a StepOnePlus™Real-Time PCR System (Applied Biosystems). The mRNA levels of octamer-binding transcription factor 4 (Oct4), kruppel-like factor 4 (KLF4), Nanog homeobox (Nanog), Tenascin C, collagen type I alpha-1 (COL1A1), and vascular endothelial growth factor (VEGF) were assessed. Somatostatin-14 peptide (S14) was used as the housekeeping gene in quantitative analysis. Primer sequences: S14, forward 5′-GTGTGACTGGTGGGATGAAGG-3′ and reverse 5′-TTGATGTGTAGGGCGGTGATAC-3′; Oct4, forward 5′-AGGAGAAGCTGGAGCAAAA-3′ and reverse 5′-GGTCGAATACCTTCCCAAA-3′; KLF4, forward 5′-GACTTCCCCCAGTGCTTC-3′ and reverse 5′-CGTTGAACTCCTCGGTCTC-3′; Nanog, forward 5′-GGTCCCAGTCAAGAAACAGA-3′ and reverse 5′-GAGGTTCAGGATGTTGGAGA-3′; Tenascin C, forward 5′-CGGGGCTATAGAACACCAGT-3′ and reverse 5′-AACATTTAAGTTTCCAATTTCAGGTT-3′; COL1A1, forward 5′-GGGATTCCCTGGACCTAAAG-3′ and reverse 5′-GGAACACCTCGCTCTCCA-3′; VEGF, forward 5′-CAACATCACCATGCAGATTATGC-3′ and reverse 5′-TCGGCTTGTCACATTTTTCTTGT-3′.

Amplification protocol: an initial denaturation at 95 °C for 3 min, followed by 40 cycles of 5 s each at 95 °C and 30 s at 57 °C. Relative quantification of target genes was performed in triplicates, analyzed using the 2^−ΔΔCt^ method and normalized to the corresponding S14 values.

### Statistical analysis

Statistical analysis was performed using GraphPad Prism v 6.0 software (GraphPad Software Inc.). Data were typical results from three replicate experiments for each of the four patients-derived cell lines, and were expressed as the mean ± standard deviation (SD). Paired comparisons were performed by two-tailed *t* test. When data was not normally distributed, the Wilcoxon matched-paired test was performed. The significance level was set at *p* value lower than 0.05.

## Results

To mimic the standard PST®-treatment procedure on patients’ rotator cuff (Fig. 2a), hTSCs cells were cultured in 150 mm^2^ dishes, placed for 1 h inside the PST® solenoid (Fig. 2b), and then returned to the CO_2_ incubator (Fig. 2c), as described in the Methods. Control hTSCs were kept outside the incubator for 1 h during the PST®-treatment time.

### Isolation, characterization, and culture of hTSCs

Isolation of hTSCs was performed according to the protocol described in the Methods section and summarized in Fig. 1a.

hTSCs were cultured to passage three, and then subjected to immunophenotyping by flow cytometry, revealing positivity for mesenchymal antigens CD73, CD90, CD105, CD166, CD106, and CD146 and negativity for hematopoietic antigens, CD19, CD34, CD45, and HLA-DR, as expected (Additional file 1: Figure S1A). As shown in Additional file 1: Figure S1B, hDFs were phenotypically characterized by flow cytometry as controls for comparison with hTSCs, revealing that the immunophenotype of hDFs does not significantly differ from that of hTSCs.

Both cell populations resulted positive for mesenchymal lineage markers like CD73, CD90 and CD105, and negative for hematopoietic lineage markers like CD19, CD34, CD45 and HLA-DR (Additional file 1: Figure S1).

To confirm their stemness, isolated hTSCs were induced to differentiate in vitro toward adipocytes, osteoblasts, chondrocytes by treatment with the proper differentiation media. Results confirmed that hTSCs could be efficiently induced to differentiate towards these cell phenotypes (Fig. 1b).

Conversely, analysis of stem cell markers Oct4, KLF4 and Nanog, measured by quantitative Real-Time PCR, confirmed the expression of the genes in hTSCs, while hDFs showed almost undetectable levels, as expected for terminally differentiated cells (Fig. 1c).

### Effects of PST® treatment on hTSCs morphology, proliferation, and viability

**Fig. 3:**
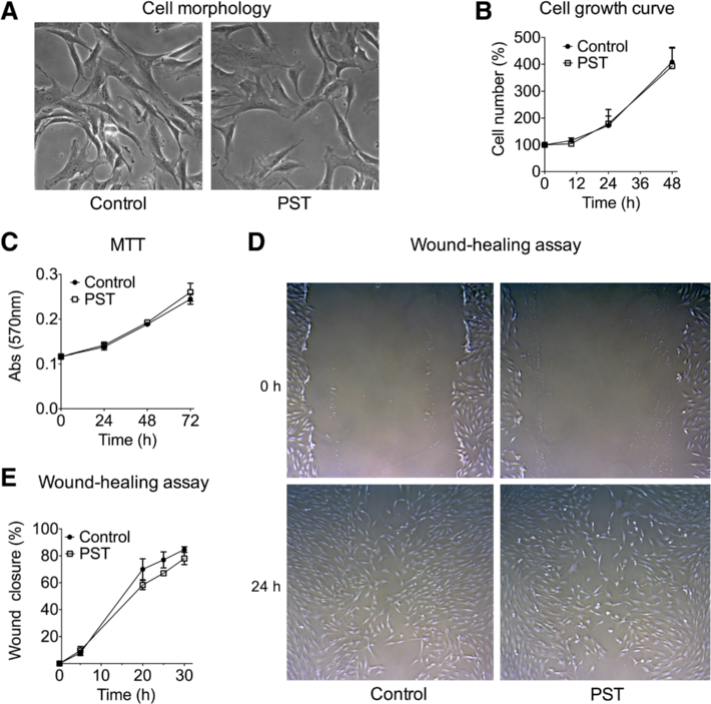
Effects of PST® treatment on hTSCs morphology, proliferation, viability, and migration. **a** Phase-contrast microphotographs (original magnification x10, at 48 h after treatment), **b** cell growth curves of hTSCs before a 1-h PST® treatment and at 10, 24, and 48 h post treatment. **c** MTT assay of hTSCs before a 1-h PST® treatment and at 24, 48, and 72 h post treatment Control cells were cultured outside the incubator for 1 h during PST® treatment. **d**, **e** Effect of PST® treatment on hTSCs migration. **d** Representative time-lapse migration images of PST and control cells. Images were acquired at 0 and 24 h in in vitro wound-healing assay. Original magnification x5. **e** The migration rate was measured by quantifying the total area of the wounded region lacking cells. The average percentages of recovered area obtained from three different experiments at 5, 10, 20, 24, and 30 h post treatment, as compared to control cells. All experiments were performed in triplicates. *Error bars* show the mean ± SD of three different experiments. Only *p*-values <0.05 are indicated, as compared to control cells

Cell morphology was analyzed by phase contrast microscopy before a 1-h PST® treatment and then 10, 24, and 48 h post treatment. Results showed no noticeable differences between PST and untreated control cells at all time points (Fig. 3a). In addition, proliferation analyses revealed an exponential cell growth in both groups, with no significant differences at all time points (Fig. 3b, *p* > 0.05). These results were confirmed by MTT cell metabolic activity assay, where no significant differences between PST and control cells could be observed at all time points (24, 48, and 72 h) (Fig. 3c).

### Effects of PST® treatment on hTSCs migration

In order to evaluate the effects of PST® treatment on the repairing capacity of hTSCs, an in vitro wound-healing assay was performed. The wound was completely closed in all conditions within 45–48 h, and PST and control cells showed a similar rate of wound closure (a representative image of PST and control hTSCs moving into the wound space is shown in Fig. 3d for both groups at 0 and 24 h after scratching). Quantitative analyses indicated no significant differences in cell migration velocity between PST and control cells at all-time points (*p* > 0.05, Fig. 3d, e).

### Effects of PST® treatment on hTSCs apoptosis

**Fig. 4:**
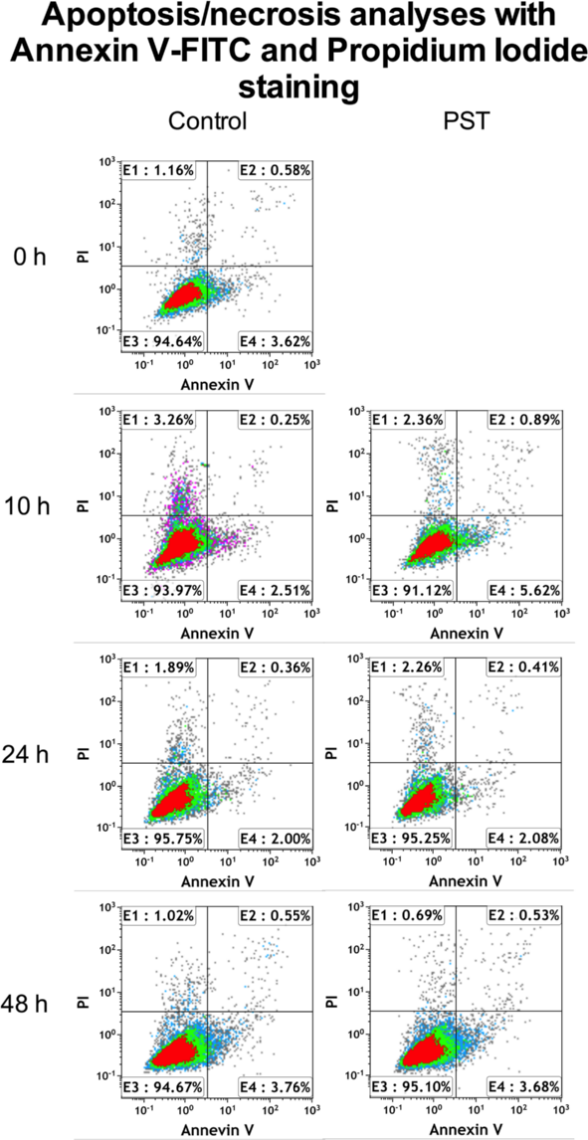
Effects of PST® treatment on apoptosis. Flow cytometric analysis of hTSCs survival rate before a 1-h PST® treatment and then 10, 24 and 48 h post treatment (*right panel*), as compared to control cells (*left panel*), through double staining with Annexin V-FITC and PI. Early apoptotic cells (Annexin V-positive/PI-negative) are localized in the *lower right* region, late apoptotic and necrotic cells (Annexin V-positive/PI-positive) in the *upper* regions, and vital cells (double negative) in the *lower left* region

Apoptosis was measured by flow cytometry before a 1-h PST® treatment and then 10, 24 and 48 h post treatment using Annexin V-FITC, and compared to control untreated cells at the same time points (Fig. 4). Results revealed no significant apoptosis in all tested samples (always below 1 %), with no statistically significant differences between PST- and control- groups.

### Effects of PST® treatment on hTSCs stem cell/tendon-related markers and VEGF

**Fig. 5:**
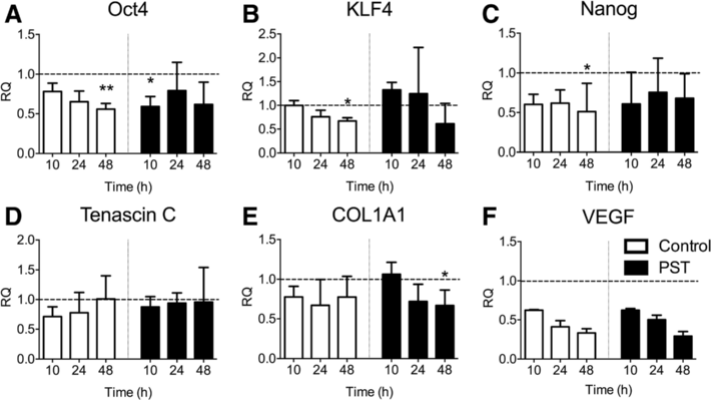
Effects of PST® treatment on stem cell marker (Oct4, KLF4, and Nanog) (**a**–**c**), tendon marker (Tenascin C and COL1A1) (**d**, **e**), and VEGF (**f**) expression by Real-Time PCR before a 1-h PST® treatment and at 10, 24, and 48 h post treatment, as compared to untreated controls. Values are expressed as fold-changes relative to untreated cells at time zero *(dotted line* set at 1). Data are expressed as means ± SD of three different experiments. *p*-values were calculated using T student test or Wilcoxon test according to data distribution. Only *p*-values <0.05 are indicated: *, *p* < 0.05; **, *p* < 0.01

The relative mRNA levels of stem cell markers Oct4, KLF4 and Nanog were measured by quantitative Real-Time PCR analyses before a 1-h PST® treatment and then 10, 24 and 48 h post treatment, and compared to the initial expression values before treatment (Fig. 5a–c). A significant reduction in stem cell marker expression at 48 h was observed in the control group, as compared to the PST cells. Oct4, KLF4 and Nanog expressions were reduced of 44 ± 7 % (*p* = 0.009), 33 ± 7 % (*p* = 0.01) and 49 ± 36 % (*p* = 0.04), respectively (Fig. 5a–c). Otherwise, stem cell marker expression of PST group was similar to that of untreated cells (*p* > 0.05). Oct4 expression at 10 h in the PST group was also significantly reduced compared to the untreated cells of 41 ± 13 % (*p* = 0.03) (Fig. 5a).

Tenascin C and COL1A1 expression levels were comparable to untreated cells (day 0) at each time point in both groups (*p* > 0.05) (Fig. 5d, e). COL1A1 expression at 48 h in the PST group was also significantly reduced compared to the untreated cells of 33 ± 19 % (*p* = 0.04) (Fig. 5e).

Analysis of the VEGF, a key marker involved in vasculogenesis and angiogenesis, by Real-Time PCR revealed no significant differences between the PST and control group in VEGF mRNA expression as compared to untreated cells (day 0) at each time point in both groups (*p* > 0.05) (Fig. 5f).

## Discussion

The possibility of activating tendon healing through electromagnetic stimulation has become increasingly popular. However, little is known about the origin of the beneficial effects that have been observed, although the possibility of stem cells involvement in the process has been often speculated. Thus the main goal of this work was to evaluate the effects of a pulsed electromagnetic field, PST®, on human tendon stem cells isolated from patients undergoing surgeries. A critical issue that had to be taken into consideration in designing this in vitro study was to maximize the cell exposure to the field. In fact, several authors have shown that the electrical field depends on the cross-sectional area of the culture media seen by the magnetic field [27–29]. Therefore, in this study, the PST® treatment was performed by placing the cell culture plate at the center of the PST® device, parallel to the ground, in order to have the magnetic field vector perpendicular to the plate surface.

The first finding of this study was that the PST® treatment (0.5–1.5 mT, 10–30 Hz, for 1 h, as in the standard treatment administered to patients) had no cytotoxic effects on hTSCs in terms of cell viability, proliferation, and migration. These results are in agreement with other studies made with different pulsed electromagnetic fields on various cell types, including human tenocytes [30–33]. However, these studies reported contrasting results on cell proliferation, which was sometimes significantly increased upon stimulation [31–35]. Nonetheless, the time of treatment, often exceeding twelve consecutive hours, could be considered rather incompatible with a realistic therapeutic application. Also, changes in cell morphology, size and orientation were occasionally reported upon pulsed magnetic field stimulation [28]. Still, in our experimental settings, hTSCs morphology was not altered by PST® exposure. Then, we tested the effects of PST® treatment on stem cell and tendon-related marker expression. Along this line, it has been observed that a single prolonged exposure to PEMFs (8 to 12 h) can positively influence the expression of tendon-specific markers (COL1A1 and SCX) in a dose-dependent manner [31, 32]. However, a 1-h PST® treatment on hTSCs had no significant effects on the expression of Tenascin C and COL1A1. Similar results were observed by Du et al. on human umbilical cord MSCs, where a 2-h treatment per day for 15 days did not cause any significant changes in the expression of several tendon and osteogenic markers, including COL1A1 [36]. On the other hand, PST® treatment seems to have a positive effect on stem cell marker expression. In fact, while the expression of stem cells markers (OCT4, Nanog, and Klf4) significantly decreased in control hTSCs, as expected during a 48 h in vitro cell culture, PST® treatment seems to maintain hTSCs in a more undifferentiated state, as no significant decrease in the same stem cell markers could be observed for 48 h after the 1-h treatment. These findings are in agreement with previous literature reports on other stem cells, which showed that the gene expression profiles regulating osteogenic and neuronal differentiation were altered after subjecting cells to an electromagnetic field treatment [37, 38]. Regarding the variability of stem cell marker expression in the PST group, this could be due to the fact that stem cells isolated from different patients were used in the study. In fact, high inter-individual differences in stem cell response could be due to a variety of parameters, including the patient’s age, co-morbidities, or the different degree of tendon injury. This could be also the cause of the discrepancies observed in the literature, where electromagnetic fields of different types were used on a variety of cells (differentiated or stem cells), which could indeed respond very differently to the treatment. Moreover, PST®, in contrast to other PEMF, uses a unique rectangular pulsed as the stimuli, which varies in amplitude and frequency [39]. Therefore, it is difficult to compare the results of the current study with previous reports, as many parameters, including the time of treatment, are different.

Finally, we tested whether the PST® treatment would increase VEGF expression, as the beneficial effects could be due to reduced inflammation and increased neovascularization. However, we could not detect any significant changes in VEGF expression upon PST® treatment on hTSCs, which doesn’t rule out that the treatment could have effects on other cell types, as endothelial cells, which are more likely to be involved in the process.

## Conclusions

In conclusion, we found that a 1-h exposure to pulsed electromagnetic field PST® did not cause any significant changes in human tendon stem cell proliferation and morphology. Nonetheless, the treatment seems to have beneficial effects on stem cell marker expression, as treated cells maintain a higher expression of these markers during the in vitro culturing, supporting the preservation of a more undifferentiated status. Clearly, the next step would be to test whether these effects could be observed in vivo in an animal model, which is currently undergoing in our laboratories.

## Abbreviations

COL1A1, collagen type I alpha-1; FBS, fetal bovine serum; hDFs, human dermal fibroblasts; hTSCs, human tendon stem cells; KLF4, kruppel-like factor 4; Oct4, octamer-binding transcription factor 4; PEMFs, pulsed electromagnetic fields; PST, pulsed signal therapy; S14, somatostatin-14 peptide; SCX, scleraxis; SD, standard deviation; VEGF, vascular endothelial growth factor; α-MEM, α-Minimal Essential Medium

## Additional file

Additional file 1: Figure S1. Phenotypic characterization of human dermal fibroblasts (hDFs) and human tendon stem cells (hTSCs) by flow cytometry. (PDF 339 kb)
