# Supplementary material for: Effects of the pulsed electromagnetic field PST® on human tendon stem cells: a controlled laboratory study
Source: BMC Complement Altern Med. 2016 Aug 18;16:293. doi: 10.1186/s12906-016-1261-3 (PMC4989537; doi:10.1186/s12906-016-1261-3)
Supplement: Additional file 1: Figure S1. — Phenotypic characterization of human dermal fibroblasts (hDFs) and human tendon stem cells (hTSCs) by flow cytometry. (PDF 339 kb) [file 12906_2016_1261_MOESM1_ESM.pdf]

Supplementary Fig.S1

A

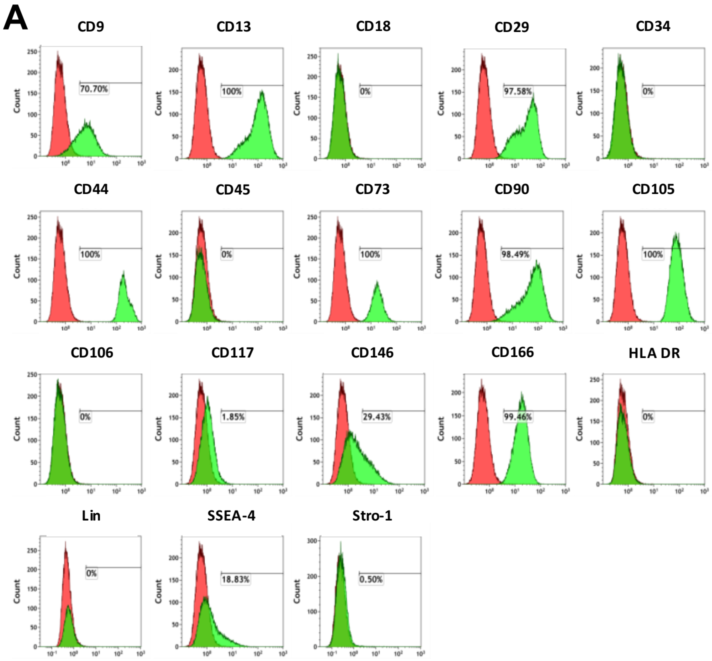

B

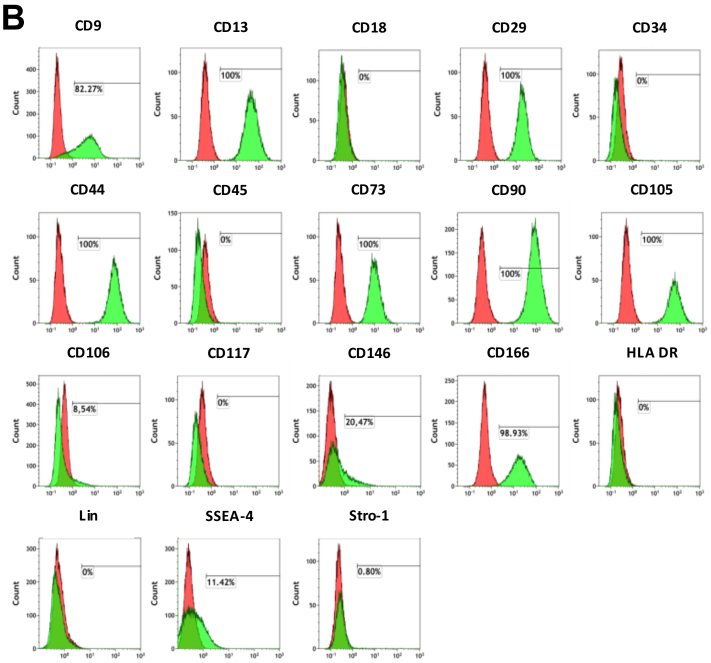

C

	hDFs	hTSCs
CD9	77,77±10,00	80,41±22,31
CD13	99,84±0,23	97,9±0,89
CD18	0±0	0±0
CD29	98,29±1,00	97,67±0,39
CD34	0±0	0,19±0,68
CD44	100±0	99,94±0,27
CD45	0±0	0±0
CD73	99,5±0,71	99,08±5,06
CD90	99,08±0,83	99,81±1,10
CD105	100±0	100±0
CD106	0±0	8,99±10,10
CD117	0,93±1,30	0,29±1,97
CD146	19,91±13,46	19,04±18,77
CD166	96,57±4,09	98,37±3,59
HLA-DR	0±0	0±0
Lineage	0±0	0±0
SSEA-4	9,415±13,31	10,02±10,53
Stro-1	0,25±0,35	4,63±8,04

Suppl.Fig.S1. Phenotypic characterization of human dermal fibroblasts (hDFs) (A) and human tendon stem cells (hTSCs) (B) by flow cytometry; representative expression panels is shown. (C) Average of marker expression levels (%±SD) of three independent experiments are reported in table.
